# Royal jelly suppresses senescence-associated secretory phenotype in senescent human epidermal keratinocytes

**DOI:** 10.1007/s11033-026-12431-4

**Published:** 2026-07-27

**Authors:** Yukie Nakagawa, Hideto Okamoto, Nobuaki Okumura

**Affiliations:** 1 Institute for Bee Products & Health Science, Yamada Bee Company, Inc., Okayama, Japan; 2 Yamada Bee Company Group Institute for Beauty Science, Yamada Bee Company, Inc., Okayama, Japan

**Keywords:** Royal jelly, Senescence-associated secretory phenotype, Epidermal keratinocytes, Skin aging, Senomorphic agent

## Abstract

**Background:**

Royal Jelly (RJ) is widely used in cosmetics for its beneficial skin effects, but its impact on cellular senescence remains unclear. Senomorphic agents that suppress the senescence-associated secretory phenotype (SASP) offer safe therapeutic strategies for skin aging. This study investigates the senomorphic effects of RJ on epidermal senescence.

**Methods and results:**

We utilized an *in vitro* replicative senescence model of normal human epidermal keratinocytes, validating our findings with cells from chronologically aged adult donors, including a 75-year-old. Results demonstrated that RJ significantly suppresses multiple SASP factors, including interleukin (IL)-6 and C-X-C motif ligand (CXCL) family members, in both models without inducing cytotoxicity. Mechanistically, RNA-sequencing revealed that senescence upregulated pro-inflammatory factors via TNF-α and NF-κB signaling pathways. RJ acts as a multitarget senomorphic agent, inhibiting these senescence-activated pathways and reducing oxidative stress.

**Conclusions:**

RJ is a potent natural senomorphic agent capable of modulating complex SASP networks. It provides valuable insights into potential strategies for mitigating skin inflammation and preserving youthfulness.

**Graphical abstract:**

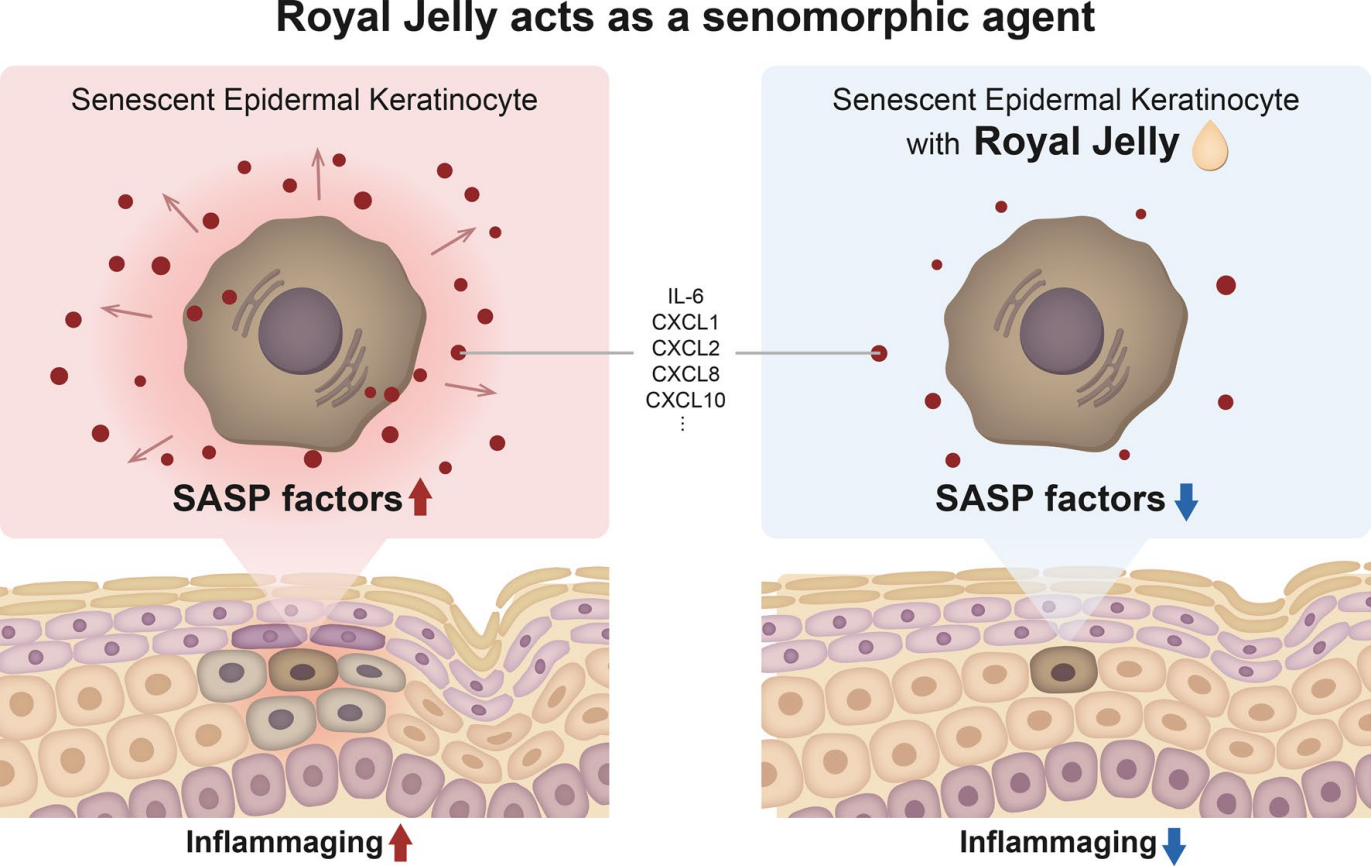

**Supplementary Information:**

The online version contains supplementary material available at 10.1007/s11033-026-12431-4.

## Introduction

Royal Jelly (RJ) is a creamy yellowish-white substance secreted by honeybees (*Apis mellifera*). RJ is rich in proteins, peptides, sugars, fatty acids, and other bioactive compounds. RJ has been widely used as an ingredient in functional foods and cosmetics [1]. Previous studies have demonstrated the benefits of RJ on the skin, including moisturization [2, 3], antioxidant [4], anti-inflammatory [5, 6], and wound healing properties [7]. Recently, continuous addition of RJ during long-term cultivation has been reported to enhance keratinocyte proliferation and reduce the proportion of Senescence-associated β-galactosidase (SA-β-gal)-positive cells [8]. RJ maintains epidermal stem cell properties by repressing senescence, making it a promising candidate for protection against skin aging. Furthermore, RJ has been reported to exert broad anti-inflammatory effects through multiple mechanisms, helping reduce inflammation in skin irritation and arthritis while also providing neuroprotection and supporting intestinal health. These diverse systemic benefits suggest that RJ may exert a protective role against cellular senescence [9, 10].

Skin aging manifests as wrinkles, dryness, and impaired barrier function [11]. These symptoms are closely associated with functional decline in keratinocytes, the primary cellular component of the outermost layer of the skin [12]. Cellular senescence is a key driver of this process [13, 14]. The phenomenon of cellular senescence was first described by Hayflick and Moorhead in 1961 [15]. Senescent keratinocytes accumulate in the skin of aged individuals and exert detrimental effects through a senescence-associated secretory phenotype (SASP) [16, 17]. The SASP is a complex secretome composed of inflammatory cytokines, chemokines, and growth factors [13, 18]. SASP factors, such as interleukin (IL)-6 and C-X-C motif ligand (CXCL) 8, reinforce senescence in an autocrine manner [18, 19]. Furthermore, SASP factors induce secondary senescence in neighboring normal keratinocytes via paracrine signaling, contributing to age-associated epidermal thinning [20]. In addition, SASP factors include plasminogen activator inhibitor-1 (PAI-1), a critical downstream target of p53 in the induction of senescence [21], and CXCL1, which promotes growth arrest via its chemokine receptor [22]. These factors are related to the stabilization and maintenance of the senescence state. Although senescent cells and their SASP are beneficial for tissue repair during wound healing when transiently present [23], their chronic accumulation drives chronic inflammation and induces or accelerates age-associated diseases [24]. The accumulation of senescent cells in the skin can accelerate whole-body aging by inducing immunosenescence and dysfunction in other organs via SASP [25]. Therefore, inhibiting SASP in keratinocytes is crucial for maintaining healthy skin and extending health span.

Senotherapeutic strategies targeting senescent cells have been developed to induce selective cell death in senescent cells, termed senolytics, and to suppress the SASP and other markers of senescence, termed senomorphics [13]. Senomorphics are agents that attenuate the deleterious effects of the SASP or suppress senescent phenotypes without inducing apoptosis. Although senolytics can promote apoptosis in senescent cells of certain cell types, they may also exhibit severe cytotoxic effects [26]. Furthermore, whether inducing senolysis in all types of senescent cells is an appropriate therapeutic strategy remains debatable because senescent cells have been shown to play crucial roles in physiological processes [26]. Senescent fibroblasts temporarily emerge in the skin during cutaneous wound healing to restrict excessive fibrosis and support normal tissue repair [27]. Therefore, the clearance of these cells may impair the regenerative capacity of the skin. In this context, senomorphics, which suppress the pro-inflammatory secretory phenotype while maintaining the beneficial features of senescent cells, represent a potentially more effective and safer therapeutic approach for skin aging [28].

However, previous studies have focused on the effects of RJ on the progression of senescence, and its potential to modulate the complex inflammatory network of established senescent cells has not been fully elucidated. In this study, we aimed to explore the senomorphic effects of RJ on senescent cells in the epidermis to evaluate its potential and practical applications. We hypothesized that RJ, a multicomponent natural agent, could comprehensively suppress various SASP factors arising from multiple signaling pathways.

## Methods

### Materials

Lyophilized raw RJ (Lot YDP-M-200225) was obtained from the Yamada Bee Company, Inc. (Okayama, Japan). RJ was standardized by the amounts of specific fatty acids, (E)-10-hydroxy-2-decenoic acid (10H2DA) and 10-hydroxydecanoic acid (10HDAA), which contained at least 3.8% 10H2DA and 0.6% 10HDAA.

### Cell culture

Normal human epidermal keratinocytes (NHEK) were purchased from PromoCell (Heidelberg, Germany) (Supplemental Table 1), and those from 75-year-old donors (NHEK 75y) were purchased from Lonza (Basel, Switzerland). NHEK were maintained in keratinocyte growth medium 2 (KGM2; PromoCell) at passages 5–7. Normal human epidermal keratinocyte progenitors from juvenile donors (HPEKs) were purchased from CELLnTEC (Bern, Switzerland). HPEKs were maintained in CnT-Prime epithelial proliferation medium (CELLnTEC) and used at passages 3–5. Replicative senescent keratinocytes were induced by long-term passaging of cells in tissue culture without any treatment. For this experiment, we used NHEK f–c at passage 20 and HPEKs at passage 10, representing fully established senescent cells. Keratinocytes were seeded in a 35-mm dish at a density of 3 × 10^4^ cells/cm^2^ and incubated for 24 h at 37 °C and 5% CO_2_. Subsequently, the cells were treated with 1 mg/mL RJ for 24 h to evaluate its acute effects on the established senescence phenotypes. Before adding it to the culture, the RJ solution was sonicated for 30 min and filtered through a 0.20 µm filter (Advantec, Tokyo, Japan).

As this study was conducted exclusively using *in vitro* cell cultures and did not involve human subjects, institutional review board approval and informed consent were not applicable.

### Senescence-associated β-galactosidase (SA-β-gal) staining

SA-β-gal activity was performed as described by Dimri et al. [29]. Keratinocytes were fixed with 4% paraformaldehyde (Nacalai Tesque, Kyoto, Japan) for 3 min and washed twice with phosphate-buffered saline (PBS). The cells were then incubated overnight at 37 °C with fresh SA-β-gal chromogenic substrate solution, 1 mg/mL 5-bromo-4-chloro-3-indolyl-β-D-galactoside, 40 mM citric acid, 5 mM potassium ferrocyanide, 5 mM potassium ferricyanide, 150 mM NaCl, and 2 mM MgCl_2_, pH 6.0. After washing, the cells were stained with Hoechst 33342 (Invitrogen, Carlsbad, USA). Images were captured using a BZ-X800 microscope (Keyence, Osaka, Japan).

### Quantitative real-time polymerase chain reaction (qPCR)

Total RNA was extracted using the NucleoSpin**®** RNA Plus kit (Macherey–Nagel, Duren, Germany). The total RNA concentration was determined using NanoDrop One (Thermo Fisher Scientific, Waltham, USA). cDNA was synthesized using ReverTra Ace**®** qPCR RT Master Mix (Toyobo, Osaka, Japan). qPCR was performed using the SYBR Green method with SsoAdvanced™ Universal SYBR® Green Supermix (Bio-Rad Laboratories, Hercules, USA) and a CFX Opus real-time PCR system (Bio-Rad). The relative expression value of each gene was calculated using the ΔΔCt method and normalized to that of *ATP5F1*. The primer sequences are presented in Table 1.

**Table 1:** Primer sequences for q-PCR

Gene name	Forward sequence (5’ → 3’)	Reverse sequence (5’ → 3’)
*p16*^*INK4a*^	GAAGGTCCCTCAGACATCCCC	CCCTGTAGGACCTTCGGTGAC
*p21*	CCGAAGTCAGTTCCTTGTGGAG	CACCTGTGAACGCAGCACAC
*IL-6*	AGACAGCCACTCACCTCTTCAG	TTCTGCCAGTGCCTCTTTGCTG
*CXCL1*	AGCTTGCCTCAATCCTGCATCC	TCCTTCAGGAACAGCCACCAGT
*CXCL2*	ACCGAAGTCATAGCCACACTCA	GGAACAGCCACCAATAAGCTTCC
*CXCL8*	GTTTTTGAAGAGGGCTGAG	TTTGCTTGAAGTTTCACTGG
*CXCL10*	GTGGCATTCAAGGAGTACCTC	GCCTTCGATTCTGGATTCAGACA
*SERPINE1*	CCAAGGACCGCAACGTGGTT	GGGGCCATGCCCTTGTCATC
*ATP5F1*	CTGTGCAGAACATGATGCGTCG	CTGTGCTTGAGCCTTCTTTGCC

### RNA-sequencing (RNA-seq)

Total RNA was extracted using a Total RNA Purification Plus Kit (Norgen Biotek, Thorold, Canada). The integrity of the total RNA samples was evaluated using a Bioanalyzer. RNA-seq libraries were constructed using the NEBNext Ultra™ II Directional RNA Library Prep Kit (New England Biolabs, Beverly, USA) and sequenced on the Illumina NovaSeq 6000 system (Illumina, San Diego, USA) in the 150 bp, paired-end configuration. Bioinformatics analysis was performed. Raw sequencing reads were trimmed using Trimmomatic (version 0.38) to remove low-quality reads and adaptors. Read quality was evaluated using FastQC (version 0.11.7), and all samples exhibited high quality, with a mean Phred score exceeding 30 at all base positions. Paired-end reads were mapped to the Homo Sapiens genome (GRCh38) using HISAT2 software (version 2.1.0). Mapped reads were counted for each annotated gene using featureCounts (version 1.6.3). Raw read counts were normalized to transcripts per million (TPMs) to account for variations in library size. Principal component analysis (PCA) was performed to assess sample clustering and detect potential outliers. Read counts were normalized using the relative log expression (RLE) method in DESeq2 (version 1.24.0) to identify differentially expressed genes (DEGs). Gene Set Enrichment Analysis (GSEA) was performed to characterize enriched Gene Ontology Biological Process (GOBP) terms associated with these DEGs [30]. In addition, Reactome pathway analysis was conducted to identify enriched biological pathways [31]. Statistical visualization, including the generation of volcano plots and heat maps, was performed in Python (version 3.11.10) using the Pandas, NumPy, SciPy, Statsmodels, Scikit-learn, and Matplotlib libraries.

### Western blot

The cells were lysed using radioimmunoprecipitation assay buffer (Nacalai Tesque) containing protease (Nacalai Tesque) and a phosphatase inhibitor cocktail (Nacalai Tesque). Protein concentration was determined using the Pierce BCA Protein Assay Kit (Thermo Fisher Scientific). Protein lysates (10 µg) were separated by 4–20% Mini-PROTEAN TGX (Bio-Rad) gel electrophoresis and transferred to a PVDF membrane using the Trans-Blot Turbo Transfer System (Bio-Rad). The membranes were washed with Tris Buffered saline (Takara Bio, Shiga, Japan) containing 0.1% Tween 20 (Nacalai Tesque) (TBST) and incubated with a blocking buffer (2% skim milk in TBST) for 1 h. Subsequently, the membranes were incubated overnight at 4 °C with primary antibodies (1:1000 dilution). The primary antibodies used for western blotting were anti-p16 (Cell Signaling Technology, Danvers, USA, #92803), anti-p21 (Cell Signaling Technology, #2947), anti-LaminB1 (Cell Signaling Technology, #13435), and anti-β-Actin (Medical & Biological Laboratories, Tokyo, Japan, PM053) antibodies. The membranes were washed with TBST and incubated with anti-rabbit IgG and horseradish peroxidase-conjugated antibodies (Cell Signaling Technology) for 1 h at room temperature. The blots were developed using a Clarity Western ECL Substrate Kit (Bio-Rad) and visualized using an image analyzer (LAS-3000 Mini; Fujifilm, Tokyo, Japan). The images were analyzed using Multi Gauge ver.3.1 software (Fujifilm).

#### Cytokine array

A protein array system was used to analyze 80 cytokines and chemokines in the conditioned medium of keratinocytes. Human Cytokine Array C5 (RayBiotech Life, Peachtree Corners, USA) was used according to the manufacturer’s protocol. The signal was quantified using Multi Gauge ver.3.1.

### Enzyme-linked immunosorbent assay (ELISA)

The levels of IL-6 in the culture supernatants were measured using a Human IL-6 Quantikine ELISA Kit (R&D Systems, Minneapolis, USA). ELISA was performed according to the manufacturer’s protocol.

### Statistical analysis

All statistical analyses were performed using GraphPad Prism 10 (GraphPad Software, Boston, USA). Data are presented as mean ± standard error of the mean (SEM). Statistical differences among three or more groups were analyzed using one-way analysis of variance (ANOVA), followed by Dunnett’s or Tukey’s multiple comparison test. For comparisons between the two groups, significance was determined using the Student’s *t*-test. Statistical significance was set at *P* < 0.05.

## Results

### Establishment of a senescence model by replicative senescence of human epidermal keratinocytes

**Fig. 1:**
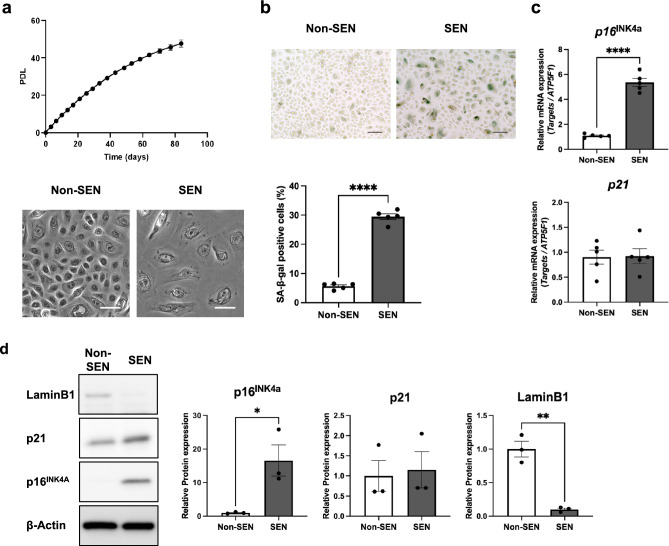
Replicative senescence in NHEK f–c. **a** Growth curves of NHEK f–c. Cells were seeded at 0.4 × 10^4^ cells/cm^2^ at each passage and cultured until sub-confluent. Scale bar = 50 µm. **b** Representative SA-β-gal stained image and the proportion of SA-β-gal-positive cells in the non-SEN and replicative senescence groups. Scale bar = 100 µm. **c** mRNA expression levels of *p16*^*INK4a*^ and *p21*. **d** Western blot analysis of p16^INK4a^, p21, and Lamin B1 protein expression. β-actin is used as a reference control. Data are presented as mean ± SEM of five independent experiments. *P < 0.05, **P < 0.01, ***P < 0.001; Student’s *t*-test. Non-SEN, non-senescent cells; SEN, Replicative senescent cells

We established an *in vitro* model of replicative senescence, as this process recapitulates the aging process in human cells. We measured the population doubling level (PDL) to characterize the replicative lifespan of NHEK f–c cells (Fig. 1a). Compared to early passage cells, the rate of proliferation gradually decreased at approximately passage 15. By passage 22 or 23, proliferation arrest was observed for approximately one month. Therefore, we used cells at passage 20, just before growth arrest, as our replicative senescence model in subsequent experiments. We used the SA-β-gal assay to evaluate the levels of senescence. This assay detects β-galactosidase activity at pH 6.0, is a widely used biomarker for senescent cells [32]. Due to this staining, the replicative senescence group showed a significantly increased proportion of SA-β-gal-positive cells compared to the non-senescence (non-sen) group (early passage cells, passage 5–7) (Fig. 1b). Furthermore, the replicative senescence group showed a significant increase in the expression of the cell cycle arrest marker p16^INK4a^ at both the mRNA and protein levels, whereas no significant changes were observed for p21 at either level (Fig. 1c, d). Conversely, the expression of Lamin B1, a nuclear membrane protein reportedly downregulated in senescent cells, decreased in the replicative senescence group. These results confirmed the establishment of cellular senescence phenotypes in this replicative model.

### Comprehensive analysis of SASP factors in the replicative senescence model

**Fig. 2:**
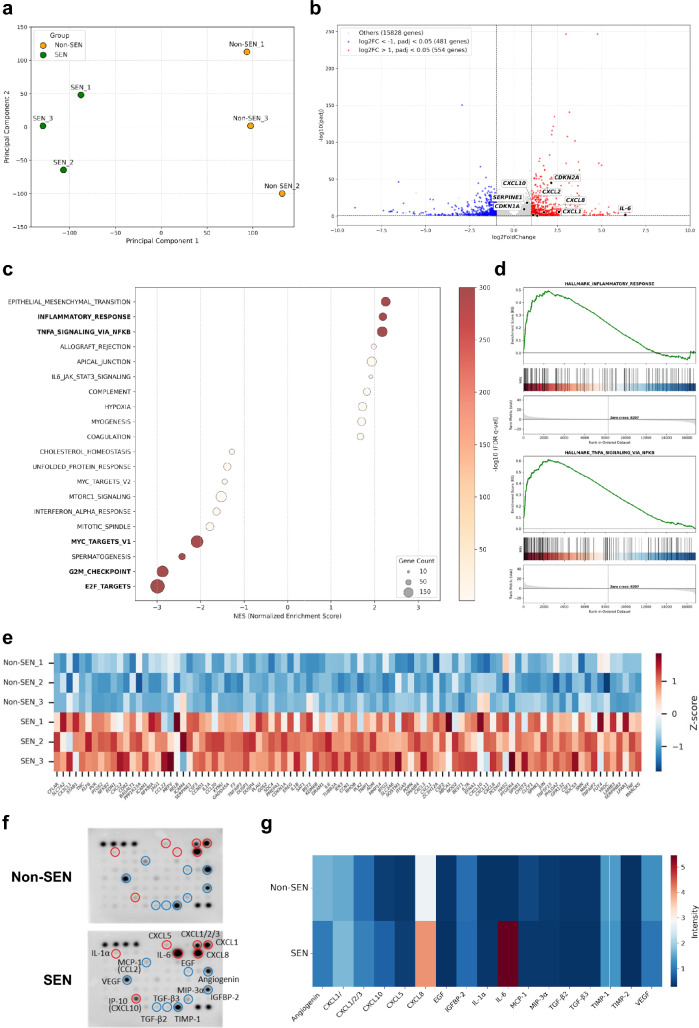
Transcriptomic and proteomic analyses of the SASP in the replicative senescence model. **a** Principal component analysis (PCA) of RNA-seq data in the replicative senescence group compared to the non-SEN group. **b** Volcano plot showing the differentially expressed genes between the non-SEN and replicative senescence groups. RNA-seq data identified 554 upregulated and 481 downregulated genes. **c** Bubble plot depicting enriched pathways, with the x-axis representing the normalized enrichment score (NES). **d** Gene set enrichment analysis (GSEA) plots for the “Inflammatory response” and “TNF-α signaling via NF-κB” showing significant enrichment in the replicative senescence group. **e** Heatmap showing significant changes in the gene expression of inflammatory response and TNF-α signaling via NF-κB pathways. **f, g** Cytokine array analysis of culture supernatants from the non-SEN and replicative senescence groups, shown as representative panels and a corresponding heatmap. Non-SEN, non-senescent cells; SEN, Replicative senescent cells

To comprehensively analyze the changes at the transcriptome level, we performed RNA-Seq analysis on our replicative senescence model, which we had confirmed exhibited senescent phenotypes. PCA of transcriptome profiles showed a clear separation between the non-sen and replicative senescence groups (Fig. 2a). We identified 1035 DEGs (|Log2(fold change)|> 1, adjusted *P* value < 0.05) between the non-sen and replicative senescence groups, comprising 554 upregulated and 481 downregulated genes (Fig. 2b). Further analysis by GSEA demonstrated that inflammatory response and tumor necrosis factor-α (TNF-α) signaling via nuclear factor kappa B (NF-κB) were strongly upregulated in the replicative senescence group. Conversely, pathways associated with E2F targets and the G2M checkpoint, which are involved in the cell cycle and DNA repair, were downregulated (Fig. 2c, d). Consistent with these findings, Reactome pathway analysis revealed that senescence-associated secretory phenotype (SASP) pathways were enriched among the upregulated genes, whereas cell cycle-related pathways were detected among the downregulated genes (Supplementary Fig. S1). Focusing on the specific components of these inflammatory signatures, we found that the expression of key SASP-related genes (*IL-1β*, *IL-6*, *CXCL1*, *CXCL2*, *CXCL3*, and *CXCL8*) was significantly elevated (Fig. 2e). Based on these findings at the mRNA level, we analyzed the secreted SASP factors in the culture supernatant using a cytokine array assay. The results confirmed increased protein secretion of SASP factors, such as IL-6 and CXCL1 (Fig. 2f, g). Collectively, these transcriptomic and proteomic findings indicate that our replicative senescence group is characterized by robust induction of inflammatory signaling and establishment of a pro-inflammatory secretory phenotype.

### Senomorphic effect of RJ on the replicative senescence model

**Fig. 3:**
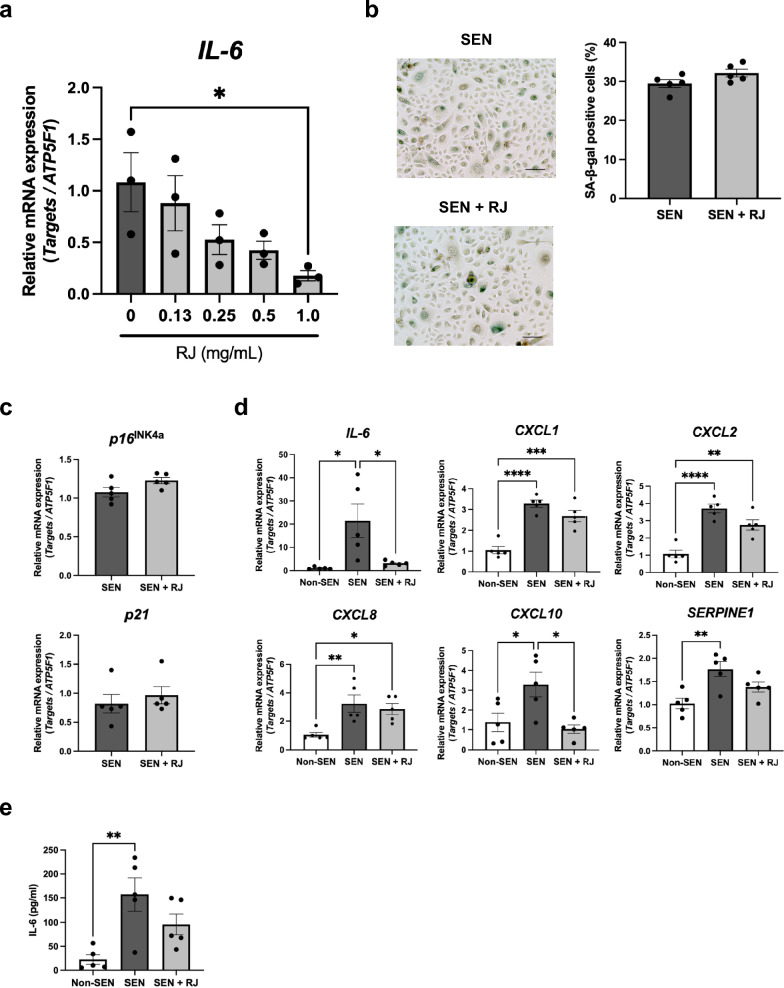
Effect of RJ on senescence markers and SASP expression in the replicative senescence model. **a** Dose-dependent mRNA expression of *IL-6* in the replicative senescence model treated with RJ. *P < 0.05; compared to RJ 0 mg/mL, one-way ANOVA and Dunnett’s multiple comparison test. **b** Representative image of SA-β-gal staining in the replicative senescence model. Scale bar = 100 µm. **c** mRNA expression of *p16*^*INK4a*^ and *p21*. **d** The mRNA expression of SASP factors. **e** IL-6 protein levels in the culture supernatants. Data are presented as mean ± SEM of 3–5 independent experiments. *P < 0.05, **P < 0.01, ***P < 0.001; according to one-way ANOVA and Tukey’s multiple comparison test. Non-SEN, non-senescent cells; SEN, Replicative senescent cells; SEN + RJ, Replicative senescent cells treated with RJ

We investigated the effects of RJ on the SASP using an established model of replicative senescence. Initially, we evaluated the cytotoxicity of RJ to determine the appropriate concentration. Although there was no statistically significant difference, cell viability was slightly increased at 2 mg/mL (Supplementary Fig. S2). Focusing on IL-6, a key SASP factor, we first performed a dose–response study to determine its optimal concentration. Treatment with RJ for 24 h decreased *IL-6* mRNA expression in a dose-dependent manner (Fig. 3a). Therefore, 1 mg/mL of RJ was used in subsequent experiments. RJ had no effect on the proportion of SA-β-gal-positive cells, which was highly upregulated in our senescent group (Fig. 3b). Furthermore, the mRNA levels of *p16*^*INK4a*^ and *p21* were not significantly altered by RJ treatment (Fig. 3c). We then assessed the effects of RJ on the five SASP factors that were upregulated in our senescent group using RNA-seq analysis. The mRNA expression levels of *IL-6* and *CXCL10* were significantly decreased following RJ treatment (Fig. 3d). However, no significant differences were observed in the mRNA expression of chemokines *CXCL1* and *CXCL2* between the two groups (*P* = 0.090 and *P* = 0.052, respectively). Consistent with the gene expression data, the secretion of IL-6 in the culture supernatant was significantly increased in our senescent group, and this increase was suppressed by RJ treatment (Fig. 3e).

### Validation of the senomorphic effect of RJ in different epidermal cell models

**Fig. 4:**
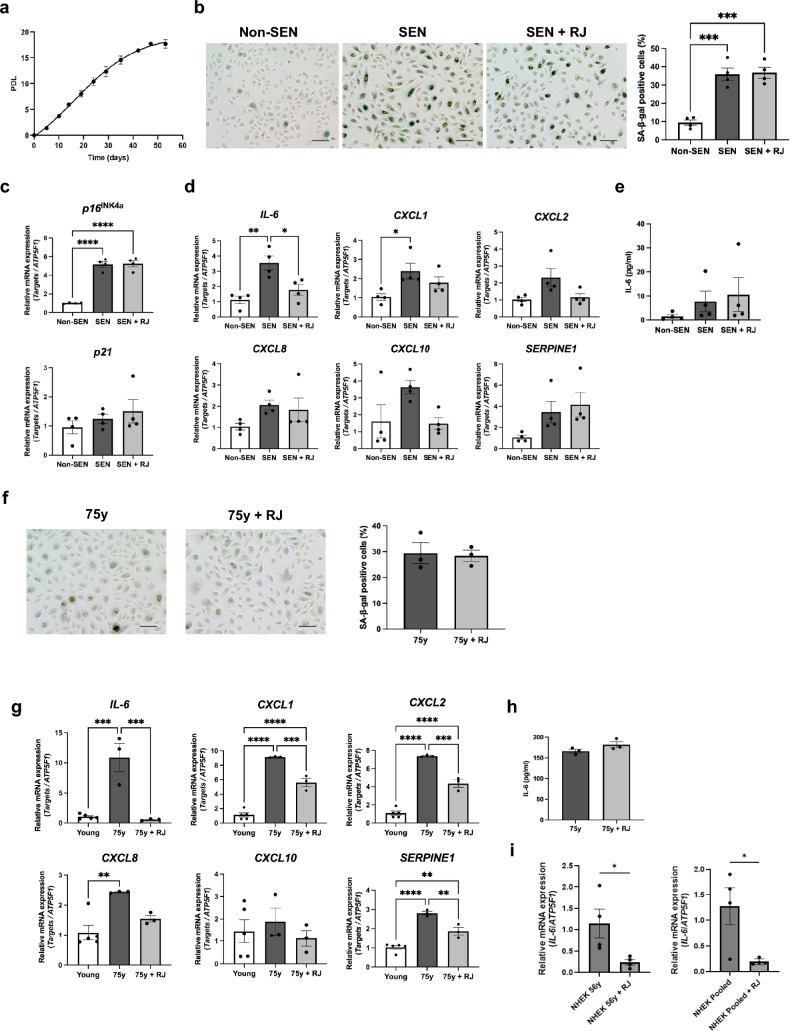
Effect of RJ on senescence markers and SASP factors in HPEKs and NHEK 75y cells. **a** Growth curve of HPEKs. **b** Representative image of SA-β-gal-stained HPEKs cells. Scale bar = 100 µm. **c** mRNA expression of *p16*^*INK4a*^ and *p21* in HPEKs. **d** The mRNA expression of SASP factors in HPEKs. **e** IL-6 protein levels in the HPEKs culture supernatants. **f** Representative SA-β-gal-stained image of NHEK 75y cells. Scale bar = 100 µm. **g** mRNA expression of SASP factors in NHEK 75y. **h** IL-6 protein levels in NHEK 75y culture supernatants. **i** mRNA expression of IL-6 in NHEK derived from adult donors (56y and Adult-pooled). Data are presented as mean ± SEM of 3–5 independent experiments. *P < 0.05, **P < 0.01, ***P < 0.001; according to one-way ANOVA and Tukey’s multiple comparison test or Student’s *t*-test. Non-SEN, non-senescent cells; SEN, Replicative senescent cells; SEN + RJ, Replicative senescent cells treated with RJ

To validate our findings in cells from different donors, we investigated the effects of RJ on a replicative senescence model established using HPEKs. The rate of HPEKs proliferation gradually decreased at passage 8 (Fig. 4a). Thus, we defined cells at passage 10 as our replicative senescence model in HPEKs for subsequent experiments. Similar to the results with NHEK f–c, the HPEKs senescent group also showed a significant increase in the proportion of SA-β-gal-positive cells and *p16*^*INK4a*^ mRNA expression. However, these senescence markers were not affected by RJ treatment (Fig. 4b, c). The mRNA expression of the SASP factors *IL-6* and *CXCL8* was significantly increased by replicative senescence in HPEKs and downregulated by RJ treatment (Fig. 4d). However, no significant differences were observed in the mRNA expression levels of *CXCL1*, *CXCL2*, and *CXCL10* (*P* = 0.057, *P* = 0.089, and *P* = 0.095, respectively). In contrast to the mRNA data, IL-6 secretion into the culture supernatant did not increase in the HPEKs senescent group and was not affected by RJ treatment (Fig. 4e).

Next, we investigated the effects of RJ on human epidermal keratinocytes derived from 75-year-old donors (NHEK 75y). The proportion of SA-β-gal-positive cells was not affected by RJ treatment, consistent with data from replicative senescence models (Fig. 4f). At the mRNA level, the expression of *IL-6*, *CXCL1*, *CXCL2*, and *SERPINE1* was significantly increased in cells from 75-year-old donors compared to that in cells from young donors, and this expression decreased following RJ treatment (Fig. 4g). *CXCL8* expression was also significantly increased in NHEK 75y. However, the reduction in *CXCL8* mRNA levels following RJ treatment was not statistically significant (*P* = 0.062). Similarly, the secretion of IL-6 was not significantly reduced (Fig. 4h; *P* = 0.081). To further account for the substantial inter-individual variability inherent to primary cell studies, we expanded our validation cohort to include one additional young donor and three additional adult donors (Supplementary Table S1). Morphological evaluation and SA-β-gal staining revealed that while the young donor cells maintained a small, highly proliferative morphology, cells from the 56y, 75y, and pooled adult donors exhibited the enlarged and flattened morphology characteristic of senescent cells. Furthermore, a higher proportion of SA-β-gal-positive cells was observed in the adult donors compared to young donors (Supplementary Fig. S3). Consistent with our earlier findings, NHEK from a 56-year-old donor (56y) exhibited significantly higher mRNA expression of the senescence marker *p16*^*INK4*^^*a*^ compared to the young donors, whereas *p21* expression remained unchanged (Supplementary Fig. S4). Regarding SASP components, we observed considerable heterogeneity among individuals. Although the 75y showed a robust and significant increase in *IL-6* expression, the newly added adult donors displayed only an increasing trend compared to the young cells. Similar individual diversity was observed for the expression of *CXCL1*, *CXCL2*, *CXCL8*, and *CXCL10*. Nevertheless, based on the morphological features and the overall expression profiles of these multiple SASP indicators and senescence markers, we determined that both the 56y and the pooled adult (Adult-pooled) donor cells exhibited distinct senescent-like phenotypes suitable for further validation. Remarkably, despite this baseline variance, RJ treatment significantly suppressed *IL-6* expression in these adult donor cells tested (Fig. 4i). These results indicate that while age-dependent SASP induction exhibits inherent individual variability, the senomorphic efficacy of RJ—particularly its ability to downregulate IL-6—is a broadly generalizable phenomenon.

## Discussion

In this study, we provided evidence that RJ functions as a potent multitarget senomorphic agent in human epidermal keratinocytes. Our results demonstrated that RJ suppresses the expression of several SASP factors involved in replicative senescence. These inhibitory effects were consistently observed in multiple independent donors, suggesting the broad senomorphic potential of RJ for both replicative and donor age-associated senescence. We also found that RJ suppressed the SASP without affecting the core hallmarks of senescent phenotypes, such as cell cycle arrest and SA-β-gal activity. As RJ exerted these suppressive effects without exhibiting senolytic activity, our findings suggest that RJ acts as a senomorphic agent.

The SASP, secreted by senescent cells that accumulate with age, causes chronic low-level inflammation associated with aging, termed inflammaging [33]. Therefore, suppression of the SASP in the epidermis, which is directly exposed to several external stressors, significantly contributes to the maintenance of skin homeostasis. Initially, we established replicative senescence in NHEK from juvenile donors as an aging epidermal model. These cells displayed a significant induction of various senescence markers, including SA-β-gal activity and p16^INK4a^ expression. Furthermore, this model showed the upregulation of SASP factors, such as IL-6 and members of the CXCL family, as determined by RNA-seq analysis and protein arrays. These expression profiles are like those reported previously for human skin aging [17, 34]. These results suggest that our replicative senescence model is related to human skin aging. However, considering its potential for future clinical translation, it is essential to verify these effects in cells that reflect chronological aging. Accordingly, we used cells derived from 75-year-old donors to evaluate the senomorphic effects in a more practical model. We found that the SASP factors that were upregulated in our replicative model, including IL-6, CXCL1, CXCL2, and SERPINE1, were also highly expressed in these elderly donor-derived cells. These findings demonstrate that our epidermal keratinocyte senescence model exhibits a senescent phenotype comparable to that of established models and simulates human skin aging.

We observed that RJ downregulated the expression of SASP factors, such as IL-6 and members of the CXCL family. These findings suggest that RJ functions as a senomorphic agent, selectively suppressing the SASP without eliminating senescent cells. This is likely because 10-hydroxy-2-decenoic acid (10H2DA), the major fatty acid in RJ, inhibits NF-κB activity [35, 36], and 10-hydroxydecanoic acid (10HDAA) contributes to anti-inflammatory effects by targeting p53 [37]. These and other bioactive components likely contributed to the suppression of SASP. A pivotal strength of this study is the validation of RJ’s efficacy in keratinocytes derived from multiple chronologically aged donors, including a 75-year-old and several other adult donors. As is common in primary human cell studies, we observed inherent inter-individual variability in the baseline expression of SASP factors, reflecting the complex and heterogeneous nature of human aging. Despite the differences between laboratory-induced replicative senescence and the complex process of chronological aging in humans, we observed a remarkable consistency in RJ’s ability to suppress SASP factors. This consistency across distinct aging models underscores their clinical translational potential beyond artificial senescence models and provides a compelling rationale for future clinical trials aimed at mitigating skin inflammation. Although RJ suppressed the gene expression of many SASP factors across several models, a significant reduction in protein secretion was confirmed only in the NHEK f–c replicative senescence model. Given that these secreted proteins are major mediators of the paracrine effects of senescent cells in the tissue microenvironment [18], further investigation is needed to fully characterize the impact of RJ on the secretome. SASP secreted by senescent epidermal cells may adversely affect epidermal stem cells and promote systemic aging through the paracrine propagation of senescence in other tissues [25, 38, 39]. Previous studies have reported that continuous addition of RJ during long-term cultivation suppresses the onset of cellular senescence and reduces the proportion of SA-β-gal positive cells [8]. While this previous finding highlights the preventive potential of long-term RJ exposure against the establishment of senescence, our present study focused on a different application: the acute senomorphic treatment of established senescent cells. Because RJ was applied to our models only for 24 h after senescence had been established, it did not reverse core senescent phenotypes like SA-β-gal activity. Instead, it selectively modulated the inflammatory secretome. Our findings suggest that RJ offers a comprehensive approach to skin aging by acting as a preventive measure against senescence and a treatment for established senescent cells. By suppressing the SASP, RJ may contribute to maintaining skin youthfulness by preserving a favorable microenvironment that supports epidermal stem cell activity.

SASP regulation is a complex process mediated by the master regulators of inflammatory responses, including NF-κB, C/EBPβ, and p38 MAPK [40–42]. In this study, RNA-seq analysis revealed that senescence-induced genes were primarily associated with the TNF-α and NF-κB signaling pathways. RJ treatment likely suppresses SASP by targeting pro-inflammatory signaling pathways, such as NF-κB signaling. Specifically, RJ has been reported to inhibit NF-κB activation and the phosphorylation of p38 and JNK, suppressing pro-inflammatory cytokines such as IL-6 [5, 43]. Moreover, RJ protects the skin from stress by inducing NQO1 expression via the Nrf2 pathway [4]. These findings suggest that RJ likely suppresses the SASP by simultaneously inhibiting senescence-activated inflammatory signaling networks, including NF-κB activation, while reducing oxidative stress. While established senomorphic agents, such as rapamycin and metformin, modulate SASP via the mTOR or AMPK pathways [26], their clinical application in skin aging still requires careful consideration. Rapamycin suppresses immune function and causes side effects such as hyperlipidemia and impaired wound healing [44]. Similarly, whereas metformin is effective in inflammatory skin diseases and suppresses the SASP in other cell types, such as endothelial cells [45, 46], its specific impact on the SASP in human skin aging remains to be fully elucidated. Unlike these single-compound agents, RJ functions as a multitarget natural senomorphic agent capable of simultaneously modulating complex inflammatory and oxidative stress networks through the synergistic actions of its diverse bioactive components. We propose that this comprehensive multitarget approach is a desirable model for future senomorphic strategies, offering a promising therapeutic means to modulate the complex inflammatory network in the skin. RJ provides an integrative approach to skin aging by preserving epidermal homeostasis and mitigating chronic low-level inflammation.

It is important to note that senomorphic activity, defined by the suppression of the SASP without eliminating senescent cells, inherently overlaps with general anti-inflammatory mechanisms because the SASP is largely driven by classical pro-inflammatory pathways. The defining characteristic of a senomorphic agent in this context is its ability to operate specifically against the endogenous secretome of senescent cells [47]. Our replicative senescence model is a genuine senescent state accompanied by profound cell cycle arrest, as evidenced by the downregulation of E2F targets and the G2M checkpoint. Furthermore, the adult and elderly primary cells used in our validation naturally exhibited elevated SASP levels driven by chronological aging, without any exogenous acute inflammatory stimuli. The ability of RJ to effectively downregulate this specific, endogenous sterile inflammation in both genuine replicative and chronological aging models strongly substantiates its classification as a potent senomorphic agent.

This study had some limitations. First, the present experiments were conducted entirely *in vitro* using specific human epidermal keratinocyte cell lines. While this model provides valuable mechanistic insights into cellular senescence, it does not fully replicate the complex, multi-cellular microenvironment of human skin *in vivo*. Second, our study focused primarily on the epidermal layer, and the potential effects of royal jelly on other skin components, such as dermal fibroblasts, remain to be fully elucidated. Therefore, further studies utilizing 3D skin equivalents, *in vivo* animal models, and ultimately clinical trials are required to validate the physiological relevance, efficacy, and safety of RJ against skin aging in humans. Furthermore, whereas we focused on the adverse effects of chronic inflammation (inflammaging), certain SASP factors can transiently contribute to tissue repair and remodeling [18]. Therefore, the long-term effects of SASP suppression by RJ on these physiological processes should be carefully evaluated in future clinical studies.

In conclusion, our data suggest that RJ exhibits potent senomorphic properties, selectively suppressing the expression of SASP factors without eliminating senescent cells. We consider RJ has the potential to serve as an anti-aging agent for maintaining skin youthfulness through its multitarget action, which simultaneously modulates the complex inflammatory networks of the SASP. Therefore, RJ could be a candidate for developing strategies for skin rejuvenation by alleviating inflammation and preserving a healthy tissue microenvironment.

## Supplementary Information

Below is the link to the electronic supplementary material.

Supplementary file1 (PDF 350 KB)

## Data Availability

The datasets generated and/or analyzed during the current study are available from the corresponding author on reasonable request.
