# Supplementary material for: Royal jelly suppresses senescence-associated secretory phenotype in senescent human epidermal keratinocytes
Source: Mol Biol Rep. 2026 Jul 27;53(1):1270. doi: 10.1007/s11033-026-12431-4 (PMC13407586; doi:10.1007/s11033-026-12431-4)
Supplement: Supplementary file 1 — Supplementary file1 (PDF 350 KB) [file 11033_2026_12431_MOESM1_ESM.pdf]

Supplementary Table S1

Supplementary Table S1 Donor information for primary human epidermal keratinocytes.

Name	Product Name	Donor Age	Supplier	Order Number	Lot Number
Young / Non-Sen	NHEK f-c pooled	4,2,5	PromoCell	C-12005	520Z012
Young Single	NHEK f-c, single donor	1	PromoCell	C-12001	474Z020.1
23y	Human cryopreserved keratinocytes	23	Biopredic International	KER110	KER110016
56y	NHEK-c adult, single donor	56	PromoCell	C-12003	451Z014.1
75y	NHEK-Adult KGM Gold	75	Lonza	00192627	18TL053546
Pooled	NHEK-c adult pooled	57,55,24	PromoCell	C-12006	473Z009.1

Supplementary Fig S1

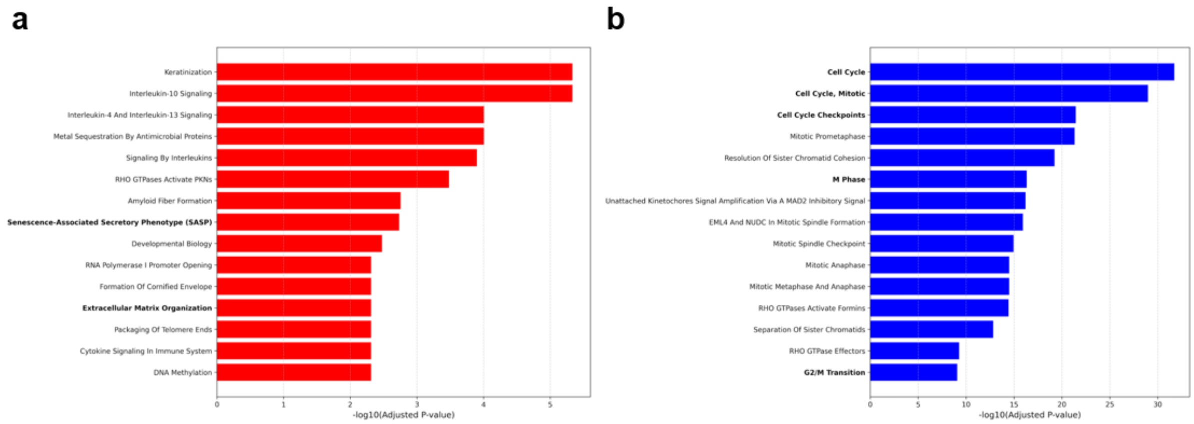

Supplementary Fig. S1 Reactome analysis of the signature differentially expressed genes (a) Ranking by adjusted *P*-value of the top 15 most highly enriched pathways for the upregulated DEGs (log_2fold change > 1), showing the enrichment of senescence-associated secretory phenotype (SASP) pathways. (b) Ranking by adjusted *P*-value of the top 15 most highly enriched pathways for the downregulated DEGs (log_2fold change < -1), highlighting the enrichment of cell cycle-related pathways.

Supplementary Fig. S2

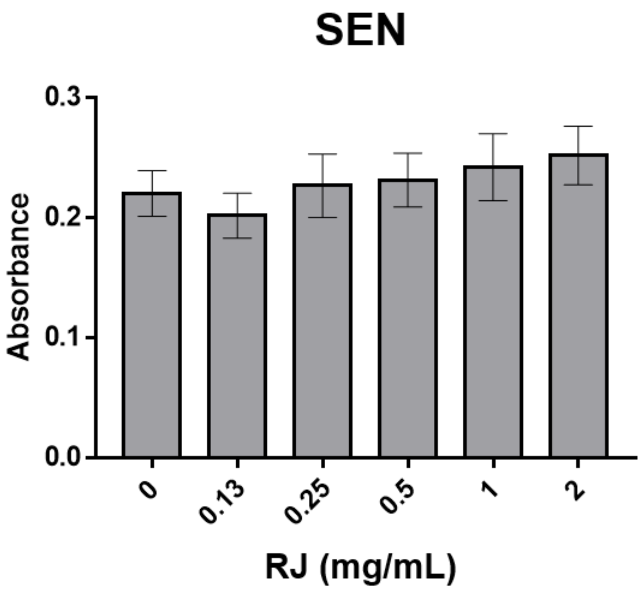

Supplementary Fig. S2 Cytotoxicity evaluation of RJ in replicative senescent cells. Cell viability of replicative senescent NHEK f-c cells treated with various concentrations of RJ for 24 h, determined by WST-8 assay. Data are presented as mean ± SEM of 3 independent experiments. Statistical significance was calculated by one-way ANOVA followed by Dunnett's multiple comparison test for comparisons against the control (RJ 0 mg/mL).

Supplementary Fig S3

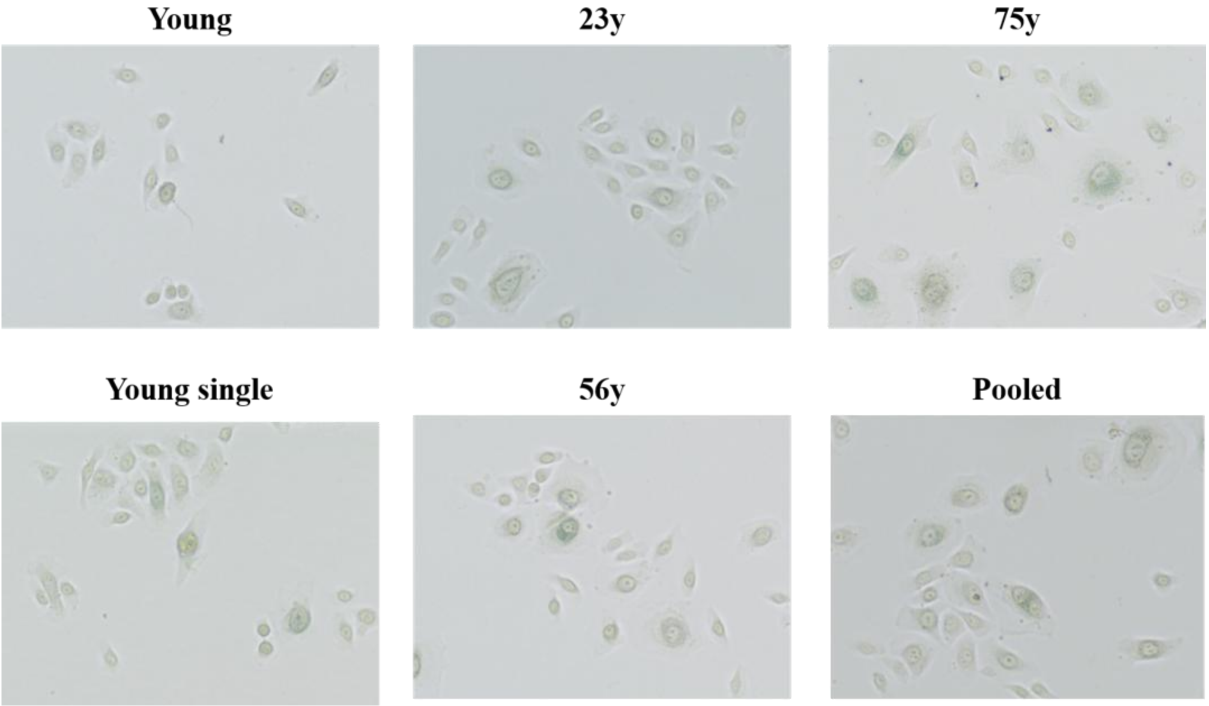

Supplementary Fig. S3 Morphological features and SA-β-gal staining in human epidermal keratinocytes from donors of different ages. Representative morphological and SA-β-gal-stained images to validate senescence phenotypes across distinct age groups. Young and Young single donor cells maintained a small, highly proliferative morphology, whereas cells from chronologically aged donors (23y, 56y, 75y, and Adult-pooled) exhibited an enlarged and flattened morphology characteristic of cellular senescence.

Supplementary Fig. S4

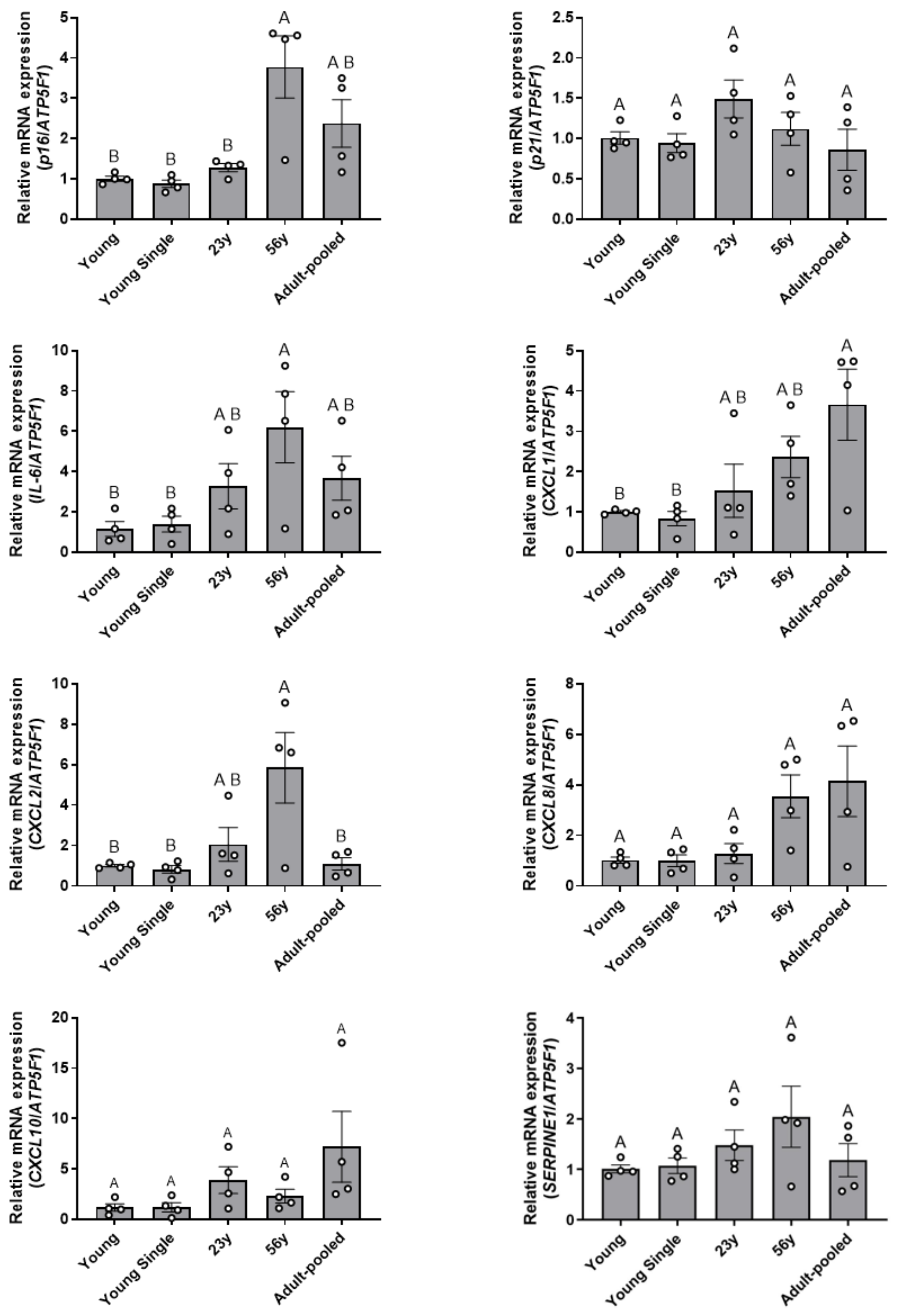

Supplementary Fig. S4 mRNA expression of cell cycle arrest markers and SASP factors in human epidermal keratinocytes from donors of different ages. Relative mRNA expression levels of cell cycle arrest markers (*p16INK4a* and *p21*) and SASP factors (*IL-6*, *CXCL1*, *CXCL2*, *CXCL8*, *CXCL10*, and *SERPINE1*) were determined by qPCR. Values were normalized to *ATP5F1*. Data are presented as mean ± SEM of 4 independent experiments. Values with different letters denote statistically significant differences ($P < 0.05$) calculated by one-way ANOVA followed by Tukey's multiple comparison test.
